# Supplementary material for: Identification and Characterization of High-Molecular-Weight Proteins Secreted by Plasmodiophora brassicae That Suppress Plant Immunity
Source: J Fungi (Basel). 2024 Jun 29;10(7):462. doi: 10.3390/jof10070462 (PMC11278463; doi:10.3390/jof10070462)
Supplement: Supplementary file 1 [file jof-10-00462-s001.zip › Supplementary Figures S1-S2.pdf]

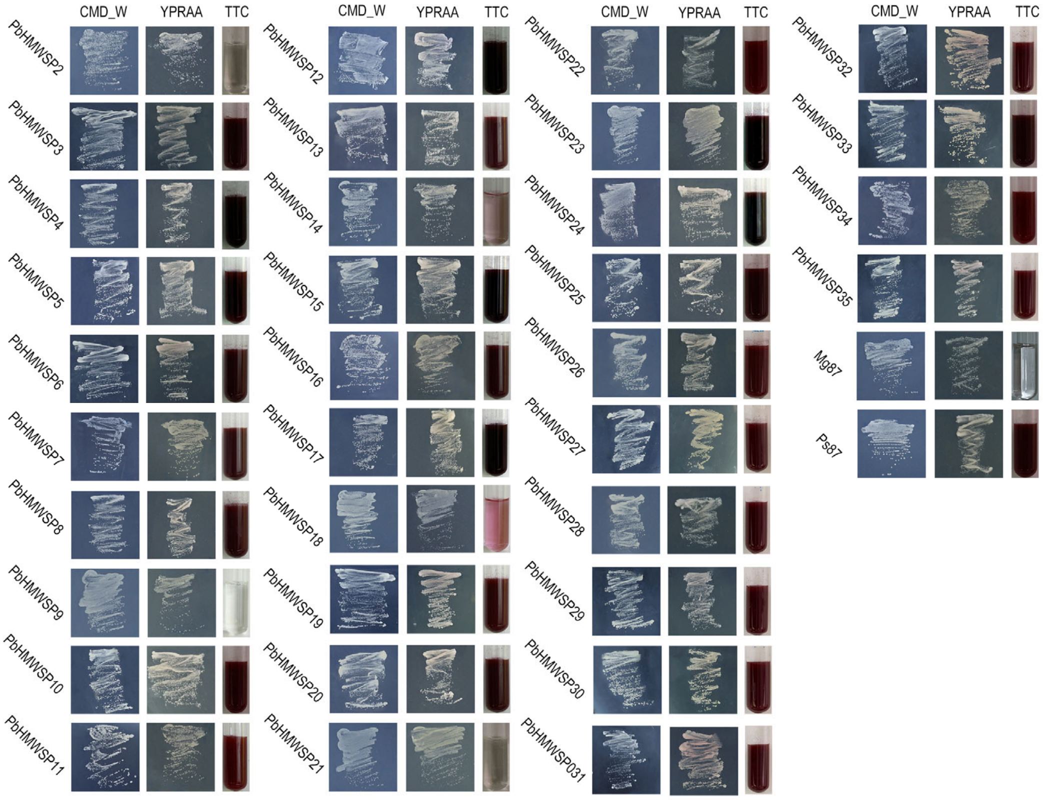

Figure S1. Functional validation of signal peptides of high molecular weight secreted proteins from *P. brassicae* by yeast signal sequence trap.

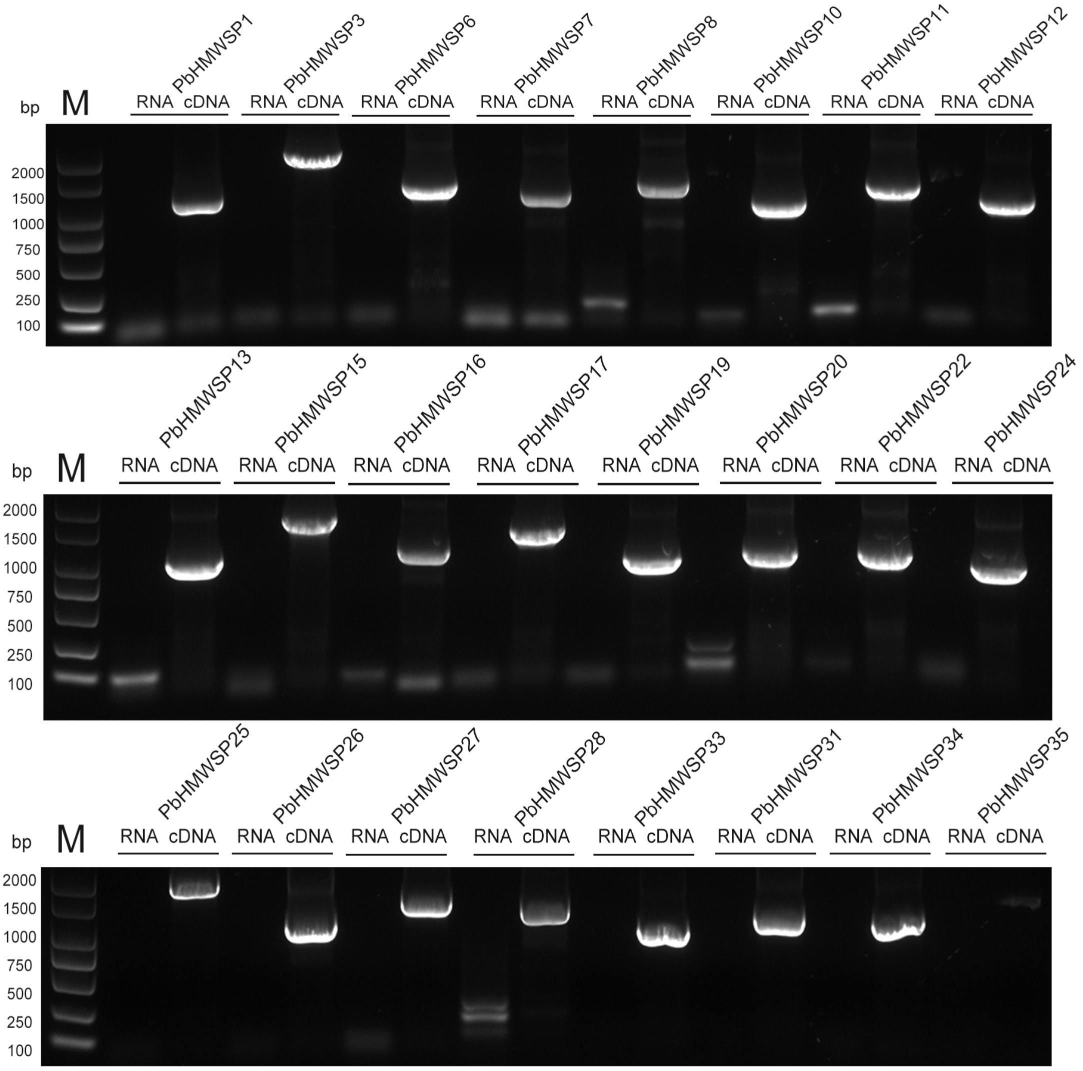

Figure S2. Detection the expression of 24 high molecular weight secreted proteins in *N. benthamiana*.
